# Eco-Friendly Catalytic Synthesis of Top Value Chemicals from Valorization of Cellulose Waste

**DOI:** 10.3390/polym15061501

**Published:** 2023-03-17

**Authors:** Onofrio Losito, Michele Casiello, Caterina Fusco, Helena Mateos Cuadrado, Antonio Monopoli, Angelo Nacci, Lucia D’Accolti

**Affiliations:** 1 Dipartimento di Chimica, Università degli Studi di Bari A. Moro, Via Orabona 4, 70126 Bari, Italy; 2 CNR-Istituto di Chimica dei Composti Organometallici (ICCOM, SS Bari), Via Orabona 4, 70126 Bari, Italy

**Keywords:** catalysis, cellulose feedstock, green chemistry, e-factor, circular economy, top chemicals

## Abstract

The total amount of cellulose from paper, wood, food, and other human activity waste produced in the EU is in the order of 900 million tons per year. This resource represents a sizable opportunity to produce renewable chemicals and energy. This paper reports, unprecedently in the literature, the usage of four different urban wastes such as cigarette butts, sanitary pant diapers, newspapers, and soybean peels as cellulose fonts to produce valuable industrial intermediates such as levulinic acid (LA), 5-acetoxymethyl-2-furaldehyde (AMF), 5-(hydroxymethyl)furfural (HMF), and furfural. The process is accomplished by the hydrothermal treatment of cellulosic waste using both Brønsted and Lewis acid catalysts such as CH_3_COOH (2.5–5.7 M), H_3_PO_4_ (15%), and Sc(OTf)_3_ (20% *w*:*w*), thus obtaining HMF (22%), AMF (38%), LA (25–46%), and furfural (22%) with good selectivity and under relatively mild conditions (T = 200 °C, time = 2 h). These final products can be employed in several chemical sectors, for example, as solvents, fuels, and for new materials as a monomer precursor. The characterization of matrices was accomplished by FTIR and LCSM analyses, demonstrating the influence of morphology on reactivity. The low e-factor values and the easy scale up render this protocol suitable for industrial applications.

## 1. Introduction

**Scheme 1:**
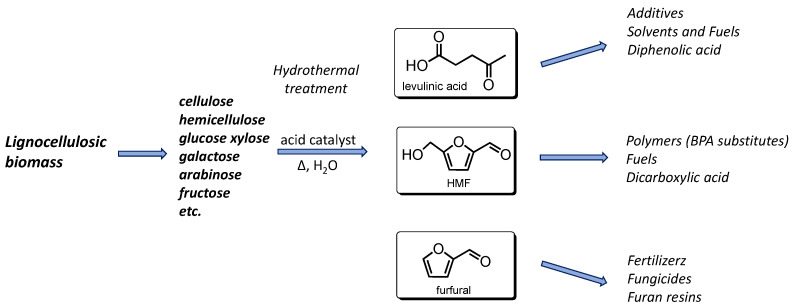
Industrial importance of furfural, HMF, and levulinic acid.

Lignocellulosic biomass is considered as the most abundant source of renewable carbon on Earth, with an estimated annual production of 2·10^11^ tons, mainly represented by residues from forestry and agriculture [1]. Given its nature as a renewable resource, lignocellulosic biomass is considered as a promising alternative to fossil sources to obtain chemicals [2,3,4] and biofuels [5,6]. Through an acid-catalyzed dehydration process, it is possible to convert the carbohydrate component of biomass (cellulose, hemicellulose, and related subunits such as glucose, xylose, galactose, arabinose, etc.) into important chemical intermediates of industrial interest such as furfural, 5-hydroxymethylfurfural (HMF), and levulinic acid (LA) (Scheme 1) [2,7].

However, the exploitation of biomass derived from dedicated crops has led to several problems such as land use changes, the increased cost of raw materials, biodiversity loss, and soil erosion. Therefore, attention has been paid to cellulosic waste and agri-food residues are currently among the most investigated materials, but their exploitation poses the drawback of subtracting these biomass fonts to the extraction of organic and inorganic micronutrients [8]. Therefore, the discovery of new wastes suitable as cellulose font has become mandatory. 

In this context, municipal waste is gaining increasing interest. Among them, cigarette butts, sanitary pants (diapers), newspapers, foods scraps (e.g., peels, grasps), and other residues have gained attention due to their large proportion, which is estimated to be in the order of megatons (Mt) per year all over the world [9]. In addition, these cellulosic matrices are still unexplored because they are considered as particularly dirty waste (cigarette butts and sanitary diapers), or have other end of life (newspaper is recycled, but in a very polluting processes) or are alternatively burned to produce energy.

The strong Brønsted acids H_2_SO_4_, HNO_3_, and HCl are the mostly efficient catalysts for the hydrothermal deconstruction of cellulose, leading to the top fine chemicals (HMF, LA, etc.), but they impose plant corrosion problems at the industrial level [10,11]. Lewis acids such as AlCl_3_ are also suitable catalysts for these processes [10], but present the drawbacks of being irritating to the respiratory system, are corrosive, moisture sensitive, and neurologically harmful [12]. 

In contrast, weak Brønsted organic acids such as CH_3_COOH or analogous less aggressive Lewis acids like scandium(III) triflate represent preferable alternatives due to the lower toxicity and corrosiveness [13]. Notably, Sc(OTf)_3_ is preferred to analogous lanthanide triflates (e.g., erbium triflate) that are very efficient in the hydrothermal conversion of cellulose, but have been declared as critical materials [14] by the new rules of EU and green chemistry [15].

Table 1 reports some representative examples of the catalytic conversion of carbohydrates into valuable chemicals such as furfural and 5-HMF, showing how CH_3_COOH and Sc(OTf)_3_ are efficient and selective compared to strong acids (entries 1, 4), even if simple monosaccharides are the preferred feedstock, while polysaccharide cellulose commonly requires more aggressive catalysts (Table 1, entry 2). 

Following our ongoing interest in developing green methods obeying circular economy principles [20,21], the aim of this work was the development of a protocol that reached two important advantages: (i) exploits municipal waste never used before (e.g., used pants and diapers, newspaper, and soybean peels) as a source of cellulose, and (ii) makes the hydrothermal treatment of cellulose more sustainable by employing less aggressive acid catalysts that are also non-critical materials such as H_3_PO_4_ [11], CH_3_COOH, and scandium(III) triflate [19], in order to obtain precious chemicals (HMF, LA, AMF, and furfural) [2,22].

## 2. Materials and Methods

Materials. Ethyl acetate (>99%) was purchased by Honeywell, phosphoric acid (85%), acetic acid (>99.8%), Sc(OTF)_3_, and all reagents and solvents were purchased from Sigma Aldrich and used without any further treatment. 

The four waste cellulosic matrices investigated, namely, cigarette butts (“Rizla + ultra slim 5.7 mm” composed of 98% cellulose acetate [11]), soybean peels (lignocellulose biomass), newspapers (composed mainly of cellulose), and Fater cellulose (diaper cellulose (composed of cellulose more 70% and 30% super absorbent polymers, personal communication of Fater group SpA) from used sanitary pants gifted by the Fater group Sp A, (Pescara, Italy), were finely chopped into small pieces. 

Acid catalysts H_3_PO_4_, CH_3_COOH, and scandium(III) triflate were dissolved into aqueous solutions. CH_3_COOH was used in two different concentrations of 4 M and 5.7 M, while phosphoric acid (H_3_PO_4_) was used at 15% *w*/*w* (1.53 M). A total of 50 mg of scandium(III) triflate was dissolved in 15 mL of water and used as a catalyst for all of the substrates in a concentration of 6.7 × 10^−3^ M.

Instrumentation. GC–MS analyses were run on a Shimadzu GLC 17-A instrument (Shimadzu, MI, Italy) using a SLB-5MS column (30 m × 0.25 mm id, film thickness of 0.25 μm). Mass spectra were performed in EI mode (70 eV) and yields of LA, HMF, AMF, and furfural were determined via GC–MS by means of calibration curves (see Supplementary Materials). ATR-FTIR spectra (Perkin elemer Waltham, MA, USA) were carried out on a Perkin-Elmer UATR-Two spectrophotometer instrument equipped with a single reflection diamond ATR crystal (refractive index of 2.4). Spectra were acquired with 32 scans in the range 4000–600 cm^−1^ by applying both the baseline and the ATR corrections. NMR spectra were recorded on a 500 MHz spectrometer: (Bruker, Milan, Italy) ^1^H NMR (500 MHz) spectra were referenced to the residual isotopic impurity of CDCl_3_ (7.25 ppm) and the ^13^C-NMR (125 MHz) spectra were referenced to 77.00 ppm. Laser confocal scanning microscopy analyses were performed with an LSM-510 confocal microscope (Zeiss, Milano, Italy).

Typical procedure for hydrothermal treatment. Weighed amounts of cellulose-based waste matrix was suspended into an aqueous solution of the acid catalyst, charged into a 300 mL stainless steel autoclave equipped with a magnetic bar and heated for the proper temperature (in the range 160–200 °C) and time (2–3 h). After cooling, the mixture was filtered and/or centrifugated to separate solid “humins”, which were dried and weighed to give a yield from 20 to 80% (depending on the reaction conditions), while the supernatant was extracted with ethyl acetate (2 × 20 mL). 

Purification procedure. The supernatant obtained from the reactions listed in Table 4, entry 1 was extracted with ethyl acetate (2 × 20 mL). Ethyl acetate, containing HMF and AMF, was washed with a 10% solution of sodium bicarbonate (2 × 20 mL) in order to remove the remaining acetic acid. The organic phase was distilled in vacuum to give the blended (HMF and AMF) product. In this case, the product was separated with a column on silica, using hexane/ethyl acetate 2:1 as the mobile phase, and giving R_f_ = 0.20 for HMF and R_f_ = 0.58 for AMF.

The reactions listed in Table 4, entries 2 and 5 and Table 5, entry 1 was repeated on the gram scale (2.5 g of substrate) in order to validate the protocol and calculate the e-factors.

Each product was isolated and characterized without further purification and the spectra were in agreement with the literature. The spectra of levulinic acid were previously reported [11] (see Supplemental Materials).

AMF (5-Acetoxymethyl-2-furaldehyde): Ref. [23] colorless oil, GC/MS (70 eV) *m*/*z* (rel. intensity). 168.30 (M^+^, 0.5), 158.30 (0.45), 142.35 (2.94), 127.20 (5.28), 126.15 (100.00), 109.10 (8.04), 97.10 (37.11), 79.05 (26.58), 53.10 (17.79), 45.00 (13.17), 43.05 (64.27). FTIR spectrum (neat) (ν, cm^−1^): 3120, 2940, 2834, 1742, 1681, 1582, 1432, 1372, 1275, 1230, 1025, 986, 945. ^1^H NMR (500 MHz, CDCl_3_) δ 9.61 (s, 1H), 7.24–7.12 (m, 1H), 6.57 (d, *J* = 3.5 Hz, 1H), 5.10 (s, 2H), 2.09 (s, 3H); ^13^C NMR (125 MHz, CDCl_3_) δ 177.81, 170.32, 155.42, 152.34, 122.48, 112.55, 57.79, 20.89.

HMF (5-(Hydroxymethyl)furfural) [24] as a dark orange oil, GC/MS (70 eV) *m*/*z* (rel. intensity): 126.95 (4.56), 125.95 (M^+^ 56.59), 108.95 (8.35), 97.95 (5.68), 96.95 (98.97), 68.95 (42.61), 53.00, (20.21), 51.00 (17.75), 43.00, (3.88), 42.00 (8.71), 41.00 (100). FTIR spectrum (neat) (ν, cm^−1^): 3122, 2931, 2844, 2718, 1683, 1370, 1280, 1191, 1072, 1023. ^1^H NMR (500 MHz, CDCl_3_) δ: 9.58 (s, 1H), 7.21 (d, *J* = 3.5 Hz, 1H), 6.51 (d, *J* = 3.5 Hz, 1H), 4.71 (s, 2H), 2.27 (s, 1H); ^13^C NMR (125 MHz, CDCl_3_) δ 177.65, 160.53, 152.36, 122.72, 109.97, 57.63.

Furfural [25] colorless oil. GC/MS (70 eV) *m*/*z* (rel. intensity): 40.05 (7.16), 41.00 (2.20), 42.00 (5.45), 51.05 (3.23), 67.00 (10.09), 95.00 (90.09), 96.00 (100.00), 97.00 (5.78); FTIR spectrum (neat) (ν, cm^−1^) 3149, 2849, 2811, 1778, 1691, 1674, 1474, 1394, 1246, 1157, 1020, ^1^H NMR (500 MHz, CDCl3): δ = 9.66 (d, *J* = 0.8 Hz, 1H), 7.69 (t, *J* = 0.8 Hz, 1H), 7.25 (dd, *J* = 3.6, 0.8 Hz, 1H), 6.60 (dd, *J* = 3.6, 1.6 Hz, 1H). ^13^C NMR (125 MHz, CDCl_3_): δ 177.93, 152.35, 148.09, 124.22, 112.57.

Calculations and data analysis. Yields in levulinic acid, HMF, AMF, and furfural were calculated based on the weight of the substrate. This yield was calculated with the ratio: product/s (g) obtained after the reaction/substrate(g) × 100. The grams of products were obtained using the GC calibration curves in the Supplementary Materials.

Regarding the literature and patent reactions, we determined the amount of waste and products (in grams) by using the conversion and molar yields.

Determination of e-factors listed in Table 6 [26].

### 2.1. E-Factor of the Reaction Listed in Table 4, Entry 2

Mass of reactants: 30.84 g of CH_3_COOH (99.8%) in 90 mL of water (the water solvent was excluded from this calculation), cigarette butts 2.5 g; total amount of reactants 30.84 g + 2.45 g = 33.29 g (considering that cigarette butts are composed of 98% of cellulose acetate).

Mass of products: 0.9771 g of 5-AMF + 0.61 g of humins = 0.15871 g

Amount of waste: (33.29 − 1.5871) g = 31.7 g

E-Factor = Amount of waste/Amount of products = 31.7/1.5871 = 19.9

### 2.2. Determination of E-Factor of the Reaction Listed in Table 4, Entry 5

Mass of reactants: 18.96 g of CH_3_COOH (99.8%) in 90 mL of water (solvent (water) was excluded from this calculation), soybean peels 2.50 g; total amount of reactants 18.96 g + 2.50 g = 21.46 g.

Mass of products: 0.51 g of Furfural + 1.05 g of humins = 1.56 g

Amount of waste: (21.46 − 1.56) g = 19.9 g

E-Factor = Amount of waste/Amount of products = 19.9/1.56 = 12.76

### 2.3. Determination of E-Factor of the Reaction Listed in Table 5, Entry 1

Mass of reactants: 0.5 g of Sc(OTf)_3_ in 90 mL of water (solvent water was excluded from this calculation), cigarette filter 2.50 g; total amount of reactants 0.5 g + 2.45 g = 2.95 g (considering that cigarette butts are composed of 98% of cellulose acetate).

Mass of products: 0.61 g of 5-HMF + 0.7 g of humins = 1.31 g

Amount of waste: (2.95 − 1.31 g) = 1.64 g

E-Factor = Amount of waste/Amount of products = 1.64/1.31 = 1.25

### 2.4. Determination of E-Factor of the Reaction Listed in Table 6, Entry 7 [14]

Mass of reactants: 12 g of CH_3_COOH (99.8%) in 60 mL of water (water solvent has been excluded from this calculation), xylose 0.6 g; total amount of reactants 12 g + 0.6 g = 12.6 g.

Mass of products: 0.307 g of furfural

Amount of waste: (12.6 − 0.307) g = 12.293 g

E-Factor = Amount of waste/Amount of products = 12.293/0.307 = 40.04

### 2.5. Determination of E-Factor of the Reaction Listed Table 6, Entry 4 [15]

Mass of reactants: 0.075 g of cellulose and 0.01 g of AlCl_3_; total amount of reactants 0.075 g + 0.01 g = 0.085 g.

Mass of products: 0.02325 g of 5-HMF

Amount of waste: (0.085 − 0.02325 g) = 0.06175 g

E-Factor = Amount of waste/Amount of products = 0.06175/0.02325 = 2.65

### 2.6. Determination of E-Factor of the Reaction Listed in Table 6, Entry 5 [17]

Mass of reactants: 0.040 g of fructose and 0.004 mg of in 2 mL of water (water solvent was excluded from this calculation); total amount of reactants 0.044 g.

Mass of products: 0.01067 g of HMF

Amount of waste: (0.044 − 0.01067 g) = 0.03333 g

E-Factor = Amount of waste/Amount of products = 0.03333/0.01067 = 3.12

### 2.7. Determination of E-Factor of the Reaction Listed in Table 6, Entry 6 [17]

Mass of reactants: 0.040 g of fructose, 2.0 g of DMSO and 0.004 g of; total amount of reactants 0.040 g + 2.0 g + 0.004 = 2.044 g.

Mass of products: 0.02332 g of HMF

Amount of waste: (2.044 − 0.02332 g) = 2.02068 g

E-Factor = Amount of waste/Amount of products = 2.02068/0.02332 = 86.65

## 3. Results and Discussion

**Characterization of the waste cellulosic matrices**. The four waste cellulosic-based matrices investigated are listed in Table 2. 

The chemical characterization of these materials was carried out using FTIR-ATR by comparing the cellulose content with that of the corresponding commercial product.

**Figure 1:**
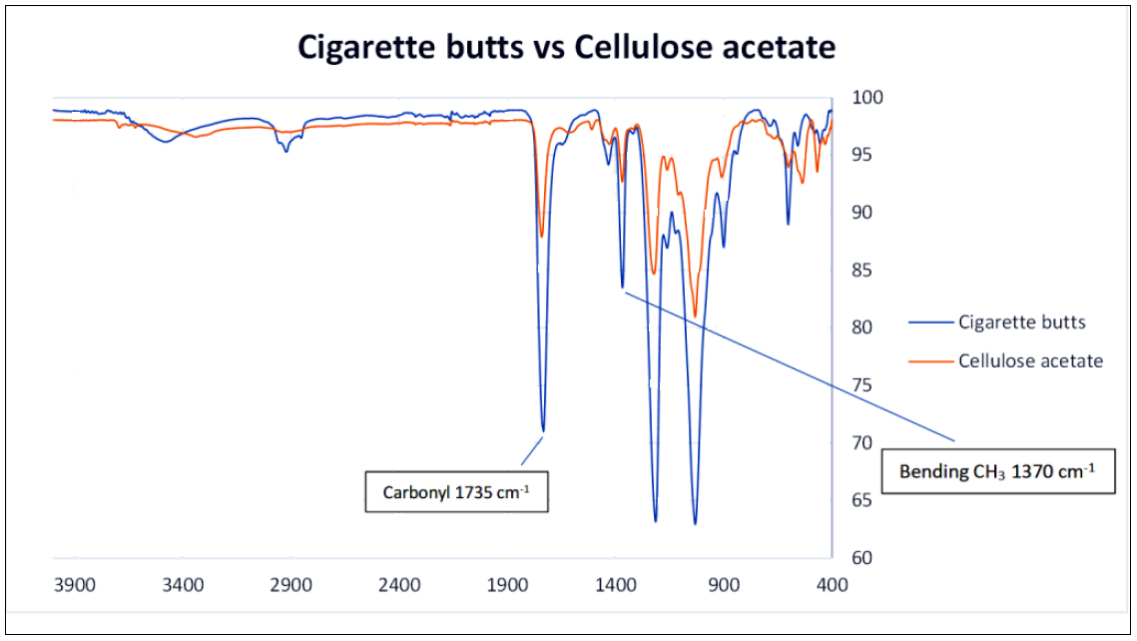
FTIR-ATR of the cellulose acetate (orange line) and cigarette butts (blue line).

**Figure 2:**
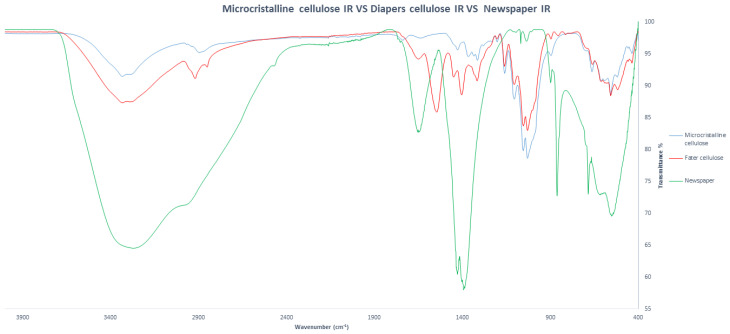
Comparison of FTIR-ATR spectra between the commercial microcrystalline cellulose (blue line) with diaper cellulose (red line) and newspaper cellulose (green line).

In addition, the cigarette butts were compared with cellulose acetate (Figure 1), while the diapers and newspaper were compared with cellulose (Figure 2).

Spectrum of cellulose acetate (Figure 1, orange line) shows the presence of three bands due to the acetyl group at 1735 cm^−1^, 1370 cm^−1^, and 1240 cm^−1^. Furthermore, the specific signals of cellulose are present in the range of 1200–1050 cm^−1^. The IR analysis of cigarette butts (Figure 1 blue line) highlights that the two profiles almost overlapped; in addition, the reduced intensity of the hydroxyls (–OH) at 3460 cm^−1^ was due to acetylation [27].

Figure 2 shows the comparison of the FTIR-ATR spectra between the commercial microcrystalline cellulose (blue line), the diaper cellulose (orange line), and the newspaper cellulose (green line). Diaper cellulose showed signals at 1648 cm^−1^ and 1545 cm^−1^, which can be attributed to the super absorbent polymer (SAP), which makes up 30% of the materials. This polymer is normally made up of polyacrylates with various degrees of polymerization that are highly hydrophilic and able to swell in the presence of water [28]. The cellulose of newspapers (green line) showed typical signals of cellulose, but also the internal signals at 1450–1350 cm^−1^ of the CH_3_ and CH_2_ stretches of the polyethylene and polypropylene coatings normally used as binders for the inks.

### Hydrothermal Tests: Synthesis of Levulinic Acid 

Preliminary experiments were devoted to extending our previous protocol for producing levulinic acid from cigarette butts [11] to the other cellulosic matrices (Table 3, entry 1). Based on the previous results, phosphoric acid was selected as the catalyst, as it allowed us to avoid the preliminary reaction with a concentration of H_2_SO_4_ suitable to disaggregate cellulose [29].

Optimized experimental conditions chosen for cigarette butts were applied to the new matrices operating on 250 mg of cellulosic material at 200 °C for 2 h. Results showed that the diapers (cellulose from sanitary pants) were more susceptible to the hydrothermal reaction than newspaper cellulose, and this latter was more reactive after a pretreatment with H_3_PO_4_ 15% for 24 h at room temperature (Table 3, entries 2–4). Notably, the reactivity of the Fater diaper cellulose did not seem to be affected by the presence of SAP.

According to the literature, the soybean peels proved to be the most reactive substrate, affording levulinic acid in a 46% of yield (Table 3, entry 5).

**Figure 3:**
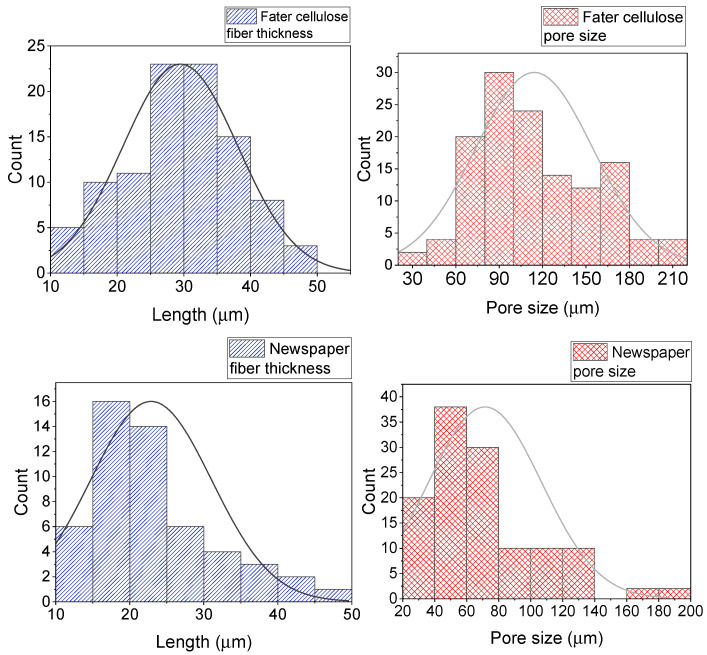
Pore size and fiber thickness distribution of the diaper cellulose and newspapers determined by LCSM analyses (at least 100 measurements).

The different reactivity of these matrices can be somehow explained based on their morphology. LCSM (laser confocal scanning microscopy) analyses of the diaper cellulose and newspapers revealed a different pore size and fiber thickness distribution, as reported in the histograms shown in Figure 3.

**Figure 4:**
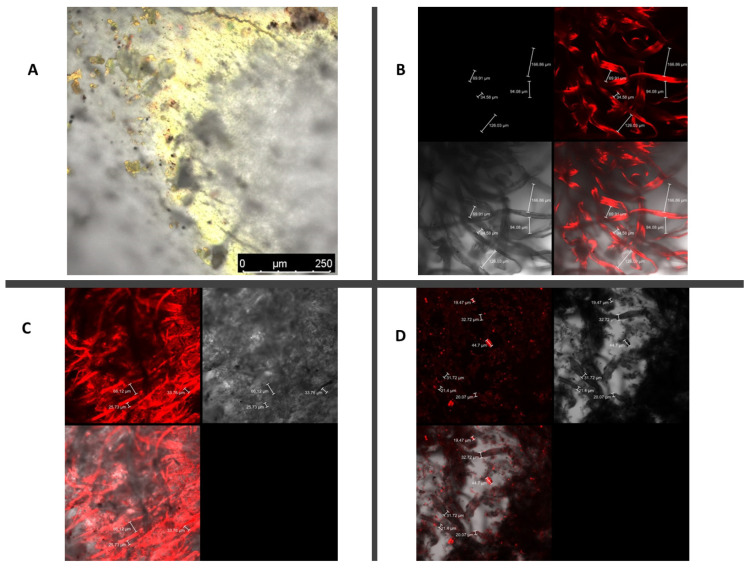
Confocal laser scanning microscopy images of (**A**) soybean peels, (**B**) diaper cellulose, (**C**) newspapers, (**D**) newspapers after treatment with H_3_PO_4_.

Newspaper fibers are denser and more compact than diaper cellulose fibers, which have larger spaces between them and are thicker (30–40 µm) than newspaper fibers (20–30 µm). This difference in morphology causes a higher reactivity and yield in the levulinic acid of diaper cellulose. Pretreatment of newspapers with phosphoric acid 15% *w*/*w* for 24 h at 25 °C caused a partial disaggregation of fibers (Figure 4D), increasing the pore sizes and consequently yielding levulinic acid to 25.6%, thus confirming the assumption that the reactivity of cellulose acetate is closely related to its morphology.

To further confirm these assumptions, an additional test was carried out by subjecting 250 mg of newspapers to acetylation with 10 mL of acetic acid and 5 mL of acetic anhydride, for 2 h at 200 °C. The completion of the acetylation reaction was confirmed by the precise overlapping of the IR spectra of the acetylated product and cigarette butts (both composed of cellulose acetate, see Supplementary Figure S1). The submission of the former to hydrothermal conditions with H_3_PO_4_ gave levulinic acid in a 22% yield, thus confirming the hypothesis in this work.

***Synthesis of furfural compounds*.** Treatment of cellulosic material with a less aggressive acid catalyst such as CH_3_COOH leads to furfural derivatives, avoiding the opening of the furanic ring. In our previous work [11], cigarette butts treated with acetic acid afforded 5-hydroxymethylfurfural (HMF) and 5-acetoxymethylfurfural (AMF) as the main products.

From these results, a dependence of reaction outcome clearly emerged from the acid catalyst loading, acetic acid was used with two different concentrations of 4 M and 5.7 M, in order to obtain furanic compounds in a selective manner [11,30].

Results in Table 4 show that treating cigarette butts with acetic acid 4 M, HMF and AMF were obtained in a ca 1:1 ratio; while increasing the acid catalyst concentration to 5.7 M, AMF was solely observed and isolated in a 38.3% yield (Table 4, entries 1 and 2).

**Scheme 2:**
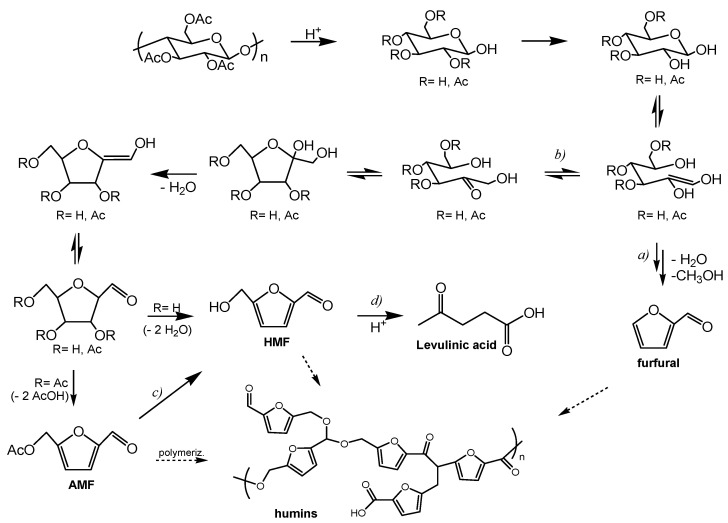
Pathways for hydrothermal hydrolysis of carbohydrates: (a) dehydration step; (b) enolization reaction; (c) deacetylation reaction; (d) hydrolysis reaction.

It should be noted that AMF is considered as a more stable and hydrophobic alternative to HMF and is therefore of great industrial importance [31]. However, only a few studies have systematically reported the synthesis of AMF from complex carbohydrates as this product is commonly obtained by the acetylation of HMF. This aspect highlights the importance of the method proposed, which enables the synthesis of AMF to a high degree of purity directly from the hydrolysis of cellulose acetate [32]. Temperature conditions affected the reaction in a profound manner. A higher heating at 220 °C solely induced the polymerization processes, leading to humins, while lower temperatures did not give rise to any reaction, even with long reaction times (Table 4, entries 3 and 4). Similar interesting results were furnished by soybean peels (mainly composed of cellulose and hemicellulose). In this case, furfural was the sole product isolated in a ca. 21% yield, which proved to be independent on the catalyst concentration (Table 4, entries 5 and 6). However, a higher loading of CH_3_COOH (5.7 M) polymerization to humins prevailed (Table 4, entry 7). Using both newspaper and diaper cellulose matrices, we only obtained humins with a higher acetic acid concentration (5.7 M), while the same matrices resulted in being unreactive with the lower CH_3_COOH loading of 4 M (Table 4, entries 8–11). In any case, the humins can be considered as a useful product because they can be used as precursors for energy applications and for innovative carbonaceous materials [11]. All of the products that formed from the various cellulose matrices were obtained through the preliminary hydrolysis of cellulose or its acetate and the formation of the glucose monomer (Scheme 2) [11]. The formation of furfural has already been hypothesized and requires both the dehydration and loss of methanol (Scheme 2, path a) [32,33].

Of particular interest is the formation of AMF; in fact, few examples reported the synthesis of this intermediate directly from carbohydrates [31,32], while this protocol allows one to obtain AMF as the sole product using 5.7 M acetic acid as the catalyst and cigarettes butts as the feedstock. In fact, after isomerization to fructose (path b), followed by a series of deacetylations and dehydrations that give rise to aromatization, the higher concentration of acetic acid should inhibit further deacetylation at the 5-position (path c), releasing AMF as the unique product. In contrast, more diluted CH_3_COOH (4 M) should enable the hydrolysis to HMF (path c), which in turn undergoes the ring opening reaction in the presence of a stronger acid (e.g., H_3_PO_4_), affording levulinic acid (path c).

Finally, cellulosic wastes were also examined under Lewis acid catalysis conditions. For this, scandium(III) triflate was chosen, being considered as a good candidate to replace analogous lanthanide triflates, as above-mentioned [19]. The data in Table 5 show that only cigarette butts were susceptible to hydrolysis by Sc(OTf)_3_ and HMF was the sole product observed. This result demonstrates how this waste matrix is promptly reactive with a wide plethora of catalysts, thus representing one of the most interesting no-cost sources of cellulose.

***E-factors evaluation*.** The sustainability of the method proposed in this work was evaluated by means of E-factors calculated for three different reactions (Table 6, entries 1–3) and compared with representative examples of the protocols in the literature (entries 4–7, see calculation in the Materials and Methods). In particular, the result in entry 1 shows how it is possible to obtain HMF in a more selective manner (lower E-factor) than the analogous protocols reported in the literature (entries 4–6), simultaneously reaching the advantages of avoiding hazardous catalysts such as AlCl_3_ (entry 4) or directly using a cellulosic matrix instead of simple monosaccharides such as fructose (entries 5–6). Similarly, entry 2 shows the formation of furfural from soybean peels catalyzed by CH_3_COOH with a selectivity higher than the analogous reaction that uses a simple monosaccharide as the reagent (entry 7). The result in entry 3 accounts for the unprecedented specific preparation of AMF directly from cigarette butts catalyzed by acetic acid with an acceptable value of E-factor.

Finally, the data in Table 6 clearly show that the E-factors of our processes were generally lower than those of analogous patent protocols based on the same catalysts and affording the same products. Therefore, the processes proposed herein can favorably compete with the analogous ones in the literature from the sustainability standpoint. Of particular interest is the comparison between the Lewis acid catalysts Sc(OTf)_3_ and AlCl_3_, which are good sustainable candidates for industry due to their very low E-factor values (1.25 and 2.65, respectively). However, unlike AlCl_3_, scandium(III) triflate is not corrosive, does not react violently with water, and is not irritating to the respiratory system; for these reasons, it is more suitable for industrial applications.

## 4. Conclusions

**Scheme 3:**
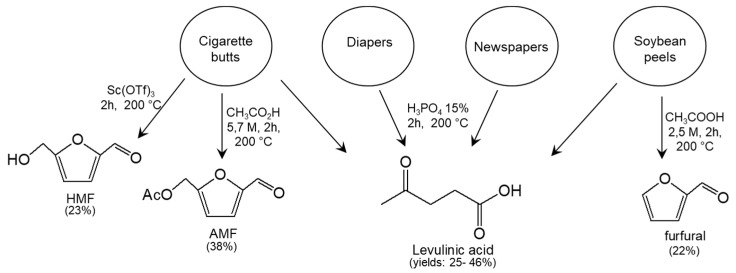
Summary of the selective synthesis of AMF, HMF, levulinic acid, and furfural from cellulosic wastes realized in this work.

The results of this work allowed us to achieve the following goals: (i) a widening of the exploitation range of waste cellulosic biomass by involving new matrices unexplored until now (e.g., diaper cellulose and newspapers); (ii) the possibility of obtaining valuable chemicals from urban and industrial cellulosic wastes whose end of life has never been assessed (e.g., diaper cellulose); (iii) the opportunity of synthesizing industrial intermediates such as AMF, HMF, levulinic acid, and furfural in a highly selective manner by properly choosing both acid catalyst and reaction conditions (Scheme 3); (iv) the adherence of protocol to the European Community advises on the use of so-called “non-critical materials” as catalysts for industry (e.g., scandium(III) triflate); and (v) the setting of processes possessing very low E-factors in accordance with the green chemistry rules.

In addition, the chemical conversion into levulinic acid proposed for newspaper cellulose can be considered as a much more sustainable and convenient valorization of this waste with respect to the common recycling to which paper is subjected, which is known to have a serious environmental impact (e.g., paper bleaching).

In conclusion, the features of high sustainability, combined with the huge amount of waste feedstock available (megatons per years), which provide a no cost unlimited source of cellulose, suggest that this protocol marks a significant step forward compared to the current literature on this important issue.

In all of these perspectives, the protocol entirely follows the circular economy principles, bringing significant benefits in terms of eco-sustainability and has good potential for industrial applications.

## Figures and Tables

**Table 1:** Representative literature processes for HMF and furfural with different matrices and catalysts.

Entry	Matrix	Catalyst	Product	Yield	Ref.
1	xylose	CH_3_COOH	furfural	80% molar yield	[16]
2	cellulose	AlCl_3_	5-HMF	31% molar yield	[17]
3	fructose	HCl	5-HMF	25.5% wt	[18]
4	fructose	Sc(OTf)_3_	5-HMF	38% molar yield	[19]

**Table 2:** Waste cellulosic matrices under investigation.

Cigarette Butts	Newspapers	Diaper Cellulose	Soybean Peels
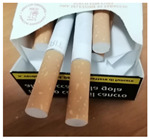	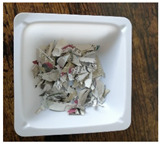	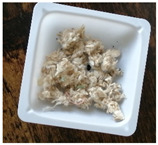	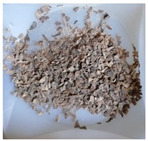

**Table 3:** Synthesis of levulinic acid from cellulosic waste ^a^.

Entry	Matrix	Catalyst ^b^	Time (h)	Temperature (°C)	Levulinic Acid ^c^ Yield% (*w*/*w*)
1 ^d^	Cigarette butts	Phosphoric acid 15% *w*/*w*	2	200	17.3
2	Diapers cellulose	Phosphoric acid 15% *w*/*w*	2	200	14.8
3	Newspapers	Phosphoric acid 15% *w*/*w*	2	200	10.0
4	Newspapers	Phosphoric acid15% *w*/*w*	2	200	25.6 ^e^
5	Soybean peels	Phosphoric acid15% *w*/*w*	2	200	46.0

^a^ General procedure: 300 mL stainless steel autoclave, matrix 250 mg, aqueous H_3_PO_4_ 15% (*w*/*w*), T = 200 °C, time = 2 h. ^b^ Volume = 15 mL, 1.53 M. ^c^ Determined by GC-MS with a calibration curve and referred to the weight of the starting matrix (250 mg). All yield values were obtained based on three replicate experiments (SD ± 2.0) (see Section 2). ^d^ Previous work result (11). ^e^ After pretreatment with H_3_PO_4_ at 25 °C for 24 h.

**Table 4:** Thermal conversion into HMF, AMF, and furfural catalyzed by acetic acid ^a^.

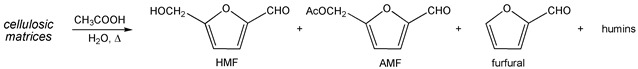					
Entry	Matrix	Catalyst CH_3_COOH (M)	Time	T (°C)	Product Yield% ^b^ (*w*/*w*)
1	Cigarette butts	4	2 h	200	HMF 21.3AMF 18.5
2	Cigarette butts	5.7	2 h	200	AMF 38.3
3	Cigarette butts	4	2 h	220	humins
4	Cigarette butts	5.7	16 h	160	n.r.
5	Soybean peels	2.5	2 h	200	furfural 21.8
6	Soybean peels	4	2 h	200	furfural 21.6
7	Soybean peels	5.7	3 h	200	humins
8	Newspapers	4	2 h	200	n.r.
9	Newspapers	5.7	2 h	200	humins
10	Diapers cellulose	4	2 h	200	n.r.
11	Diapers cellulose	5.7	2 h	200	humins

^a^ General reaction conditions: 300 mL stainless steel autoclave, matrix 250 mg, 15 mL of aqueous CH_3_COOH as the catalyst. ^b^ Referred to the substrate weight (see Section 2). All yield values were obtained based on three replicate experiments (SD ± 2.5).

**Table 5:** Hydrothermal conversion of matrices in HMF catalyzed by Sc(OTf)_3_^a^.

Entry	Substrate	Time (h)	Temperature	Products Yield% (*w*/*w*) ^b^
1	Cigarette butts	2 h	200 °C	HMF 23.2
2	Newspapers	2 h	200 °C	n.r.
3	Soybean peels	2 h	200 °C	n.r.
4	Diaper cellulose	2 h	200 °C	n.r.

^a^ General reaction conditions: 300 mL stainless steel autoclave, matrix 250 mg, catalyst 15 mL of an aqueous solution containing 50 mg of Sc(OTf)_3_ (6.7 × 10^−3^ M). ^b^ Refers to the substrate weight (see Section 2). All yield values were obtained based on three replicate experiments (SD ± 2.5).

**Table 6:** Comparison of the E-factors with the literature for representative reactions of this work.

Entry	Matrix	Catalyst	Product	Yield	E-Factor	Ref.
1	Cigarette butts	Sc(OTf)_3_	HMF	23.3% (*w*/*w*)	1.25	This work ^a^
2	Soybean peels	CH_3_COOH	furfural	21.6%(*w*/*w*)	12.76	This work ^c^
3	Cigarette butts	CH_3_COOH	AMF	38.3% (*w*/*w*)	19.90	This work ^b^
4	Cellulose	AlCl_3_	HMF	31% molar yield	2.65	[15]
5	Fructose	Sc(OTf)_3_/H_2_O	HMF	38.5% molar yield	3.12	[17] ^d^
6	Fructose	Sc(OTf)_3_/DMSO	HMF	83.3% molar yield	86.65	[17] ^e^
7	Xylose	CH_3_COOH	furfural	80% molar yield	40.04	[14]

^a^ Table 5, entry 1. ^b^ Table 4, entry 2. ^c^ Table 4, entry 5, ^d^ Reaction in water. ^e^ Reaction in DMSO.

## Data Availability

All of the data are reported in the paper and Supplementary Materials.

## Supplementary Materials

The following supporting information can be downloaded at: https://www.mdpi.com/article/10.3390/polym15061501/s1 and are available: FTIR-ATR spectra of the cigarettes butts and acetylate newspaper; supplementary characterization of furfural, HMF, AMF, and levulinic acid; calibration curve for the determination of yields.
