# Supplementary material for: Eco-Friendly Catalytic Synthesis of Top Value Chemicals from Valorization of Cellulose Waste
Source: Polymers (Basel). 2023 Mar 17;15(6):1501. doi: 10.3390/polym15061501 (PMC10053711; doi:10.3390/polym15061501)
Supplement: Supplementary file 1 [file polymers-15-01501-s001.zip › polymers-2252404-supplementary.pdf]

Supplemental Materials

Eco-Friendly catalytic synthesis of Top value chemicals from valorization of cellulose waste

Onofrio Losito [1], Michele Casiello [1], Caterina Fusco [2], Helena Mateos Cuadrado [1], Antonio Monopoli [1], Angelo Nacci [1,2] and Lucia D'Accolti [1,2,*]

1 Dipartimento di Chimica, Università degli Studi di Bari A. Moro, Via Orabona 4, 70126 Bari, Italy
2 CNR-Istituto di Chimica dei Composti Organometallici (ICCOM, SS Bari), Via Orabona 4, 70126 Bari, Italy
* Correspondence: lucia.daccolti@uniba.it

(18 pages including the cover)

Table of Contents

FTIR-ATR spectra of cigarettes butts and acetylate Newspaper p. *S2*
Supplemental characterization of furfural, HMF, AMF and levulinic acid pp. S3-S13
Calibration curve for the determination of yields pp. S14-S15

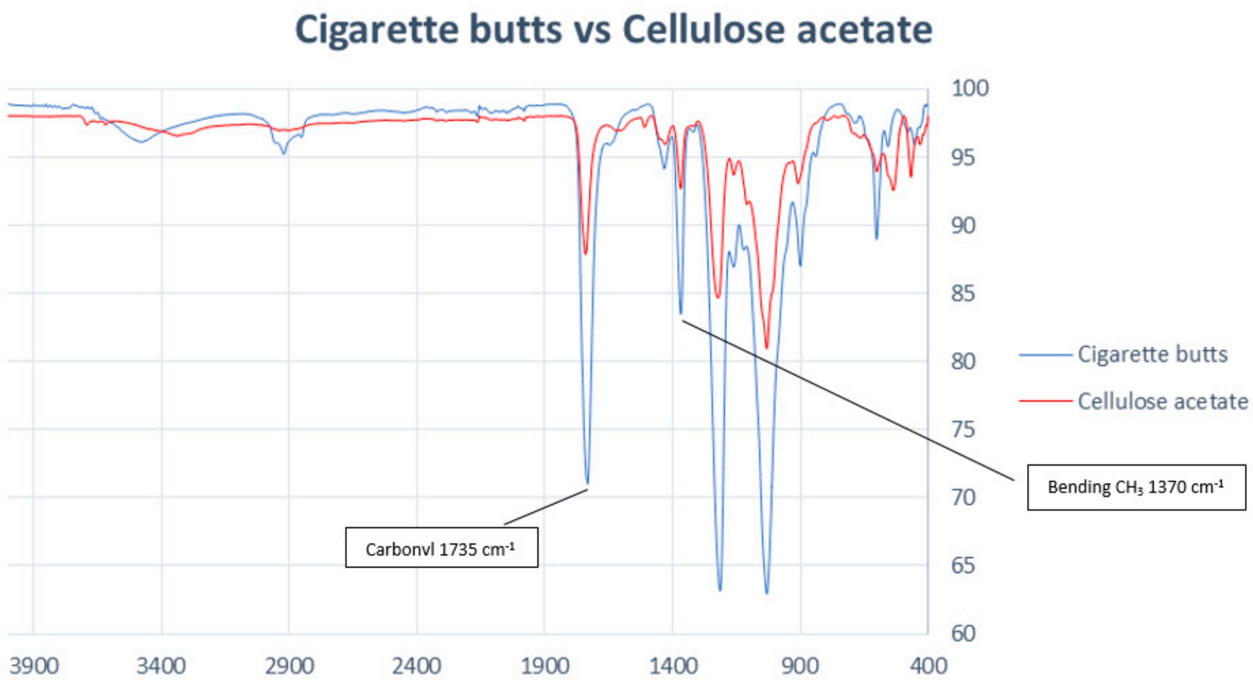

Figure S1. FTIR-ATR spectra overlying of cigarette butts and the reaction product of the acetilation reaction of news paper

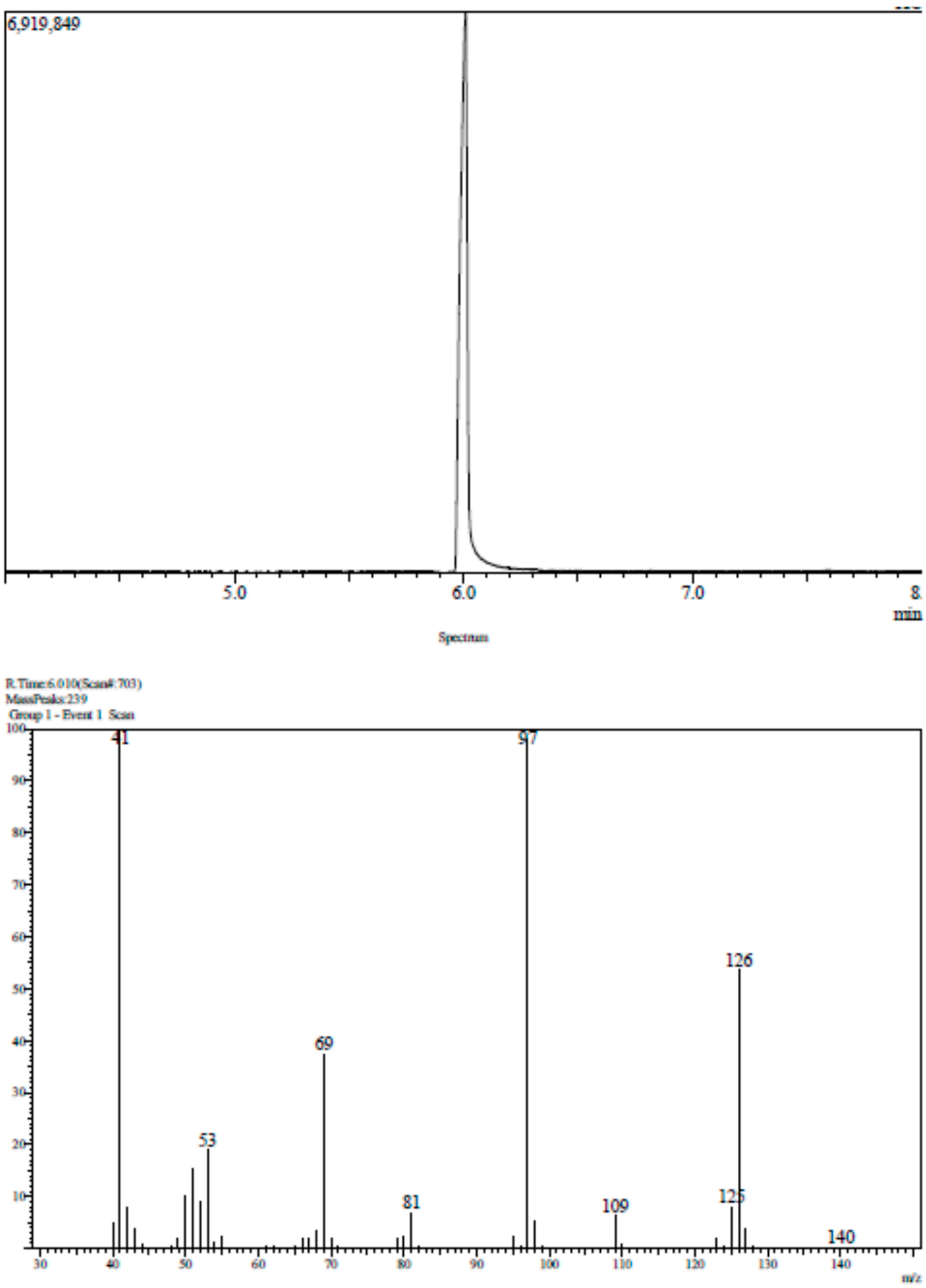

*Figure S2***.** GC/MS analysis of 5-HMF

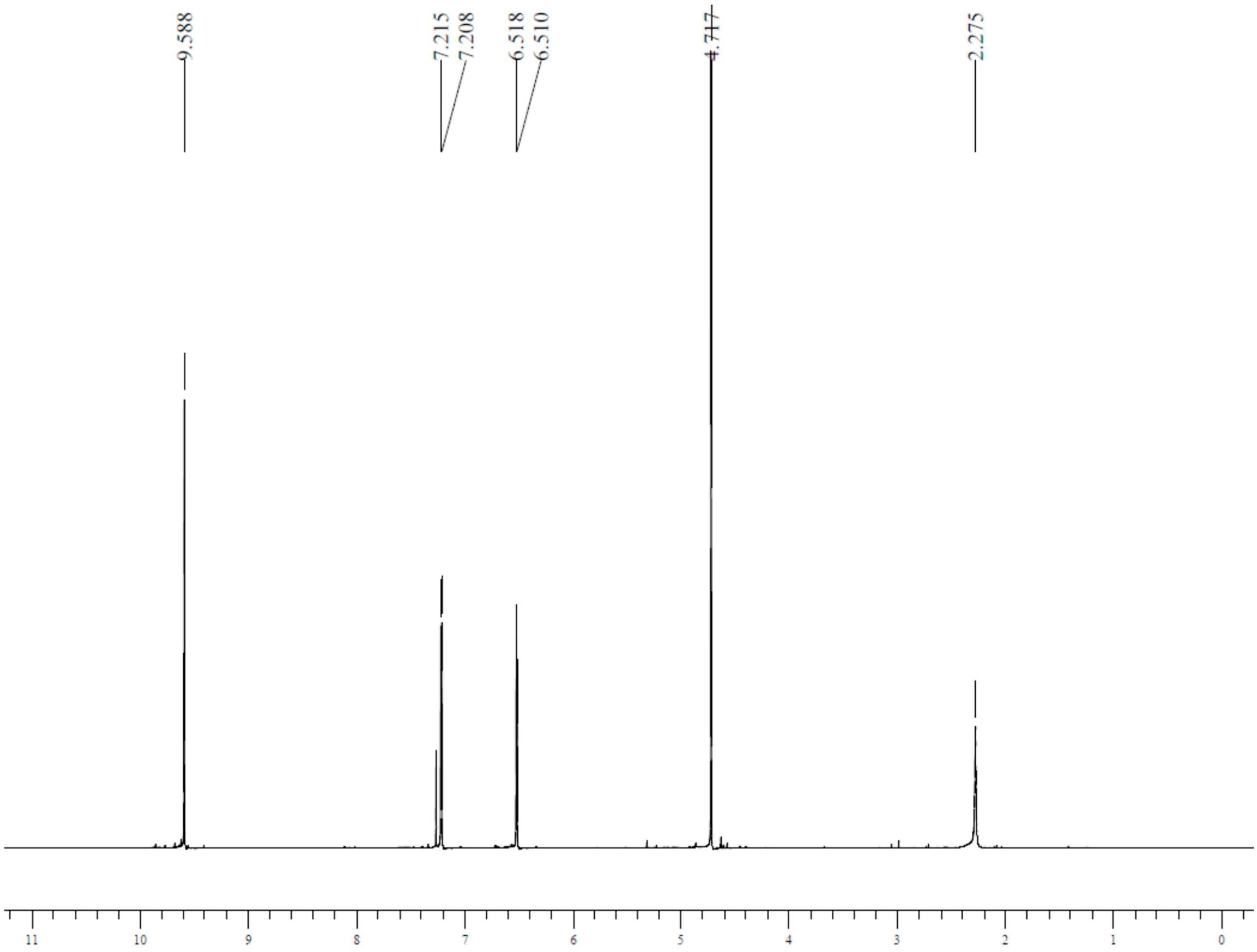

Figure S3. ^{1}H-NMR (500 MHz, $CDCl_3$) of 5-HMF

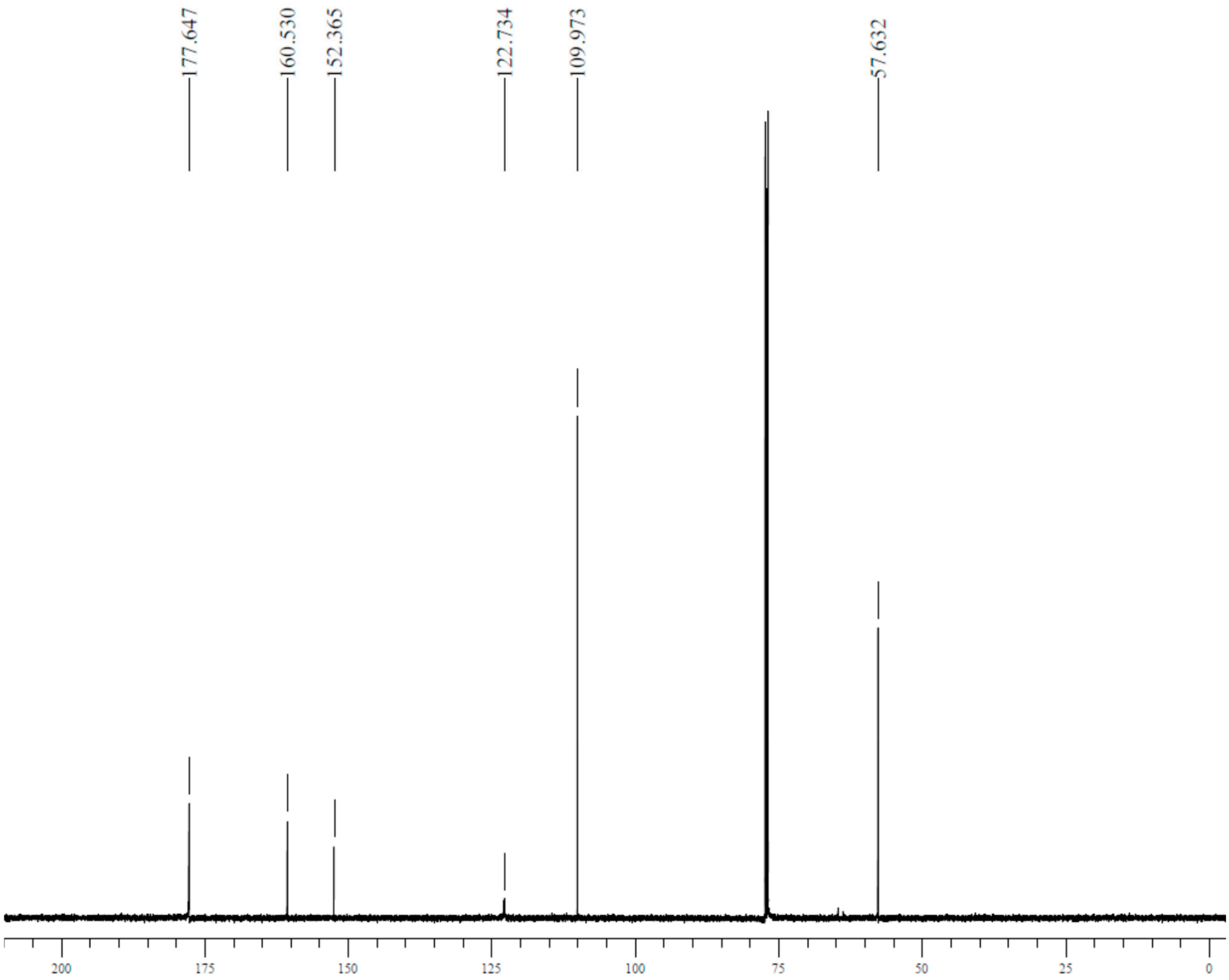

*Figure S4***.** ^{13}C-NMR (125 MHz, $CDCl_3$) of 5-HMF

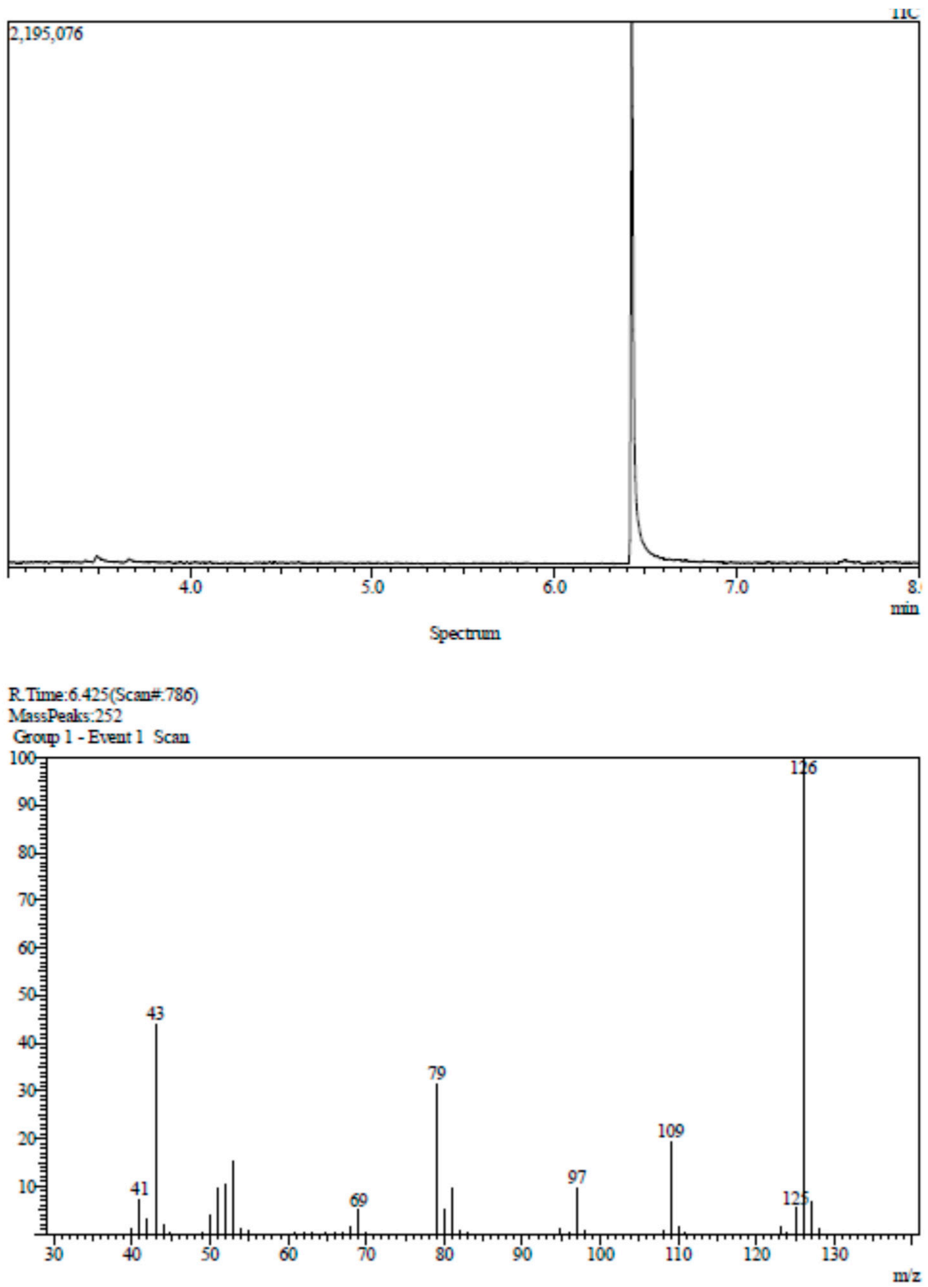

Figure S5. GC/MS analysis of 5-AMF

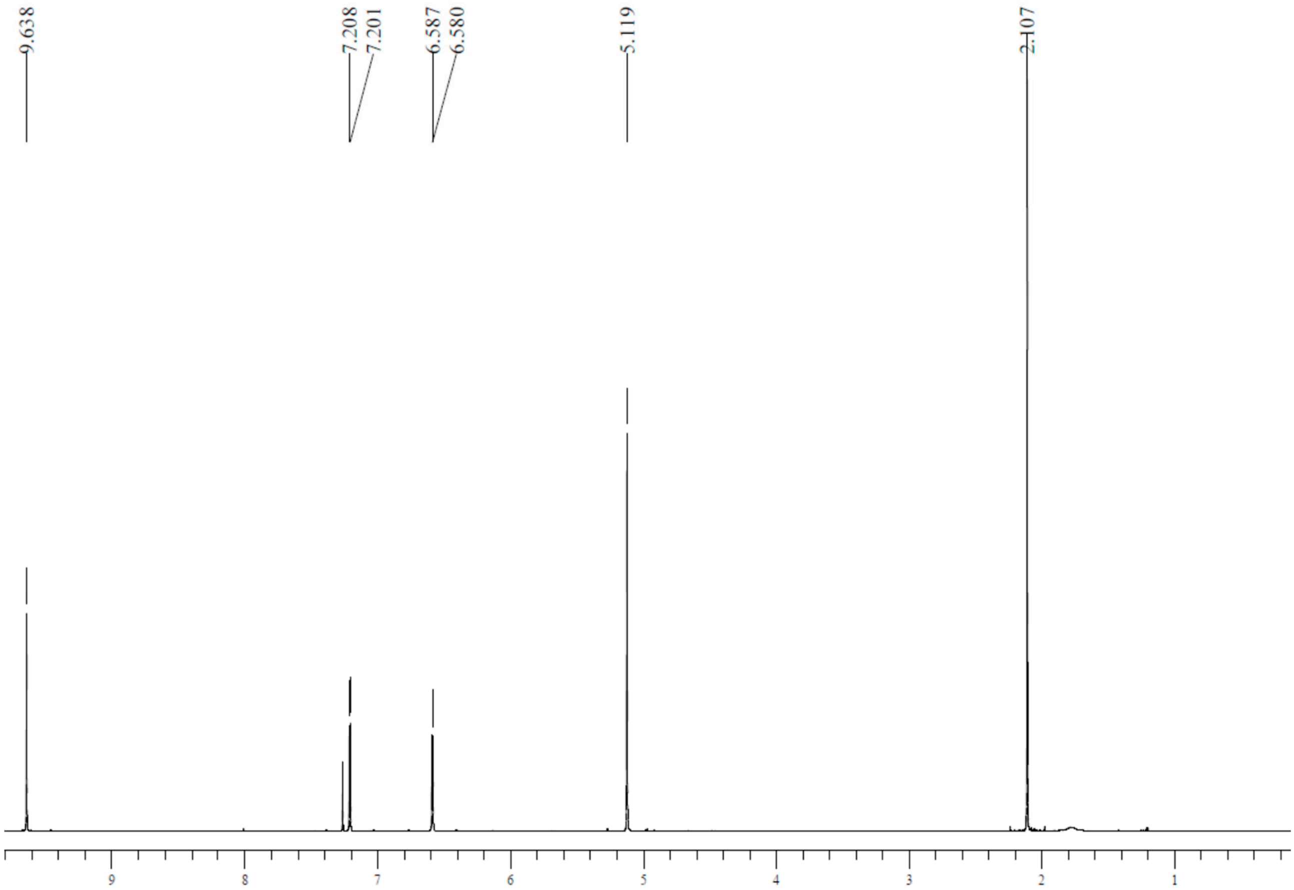

Figure S6. ^{1}H-NMR (500 MHz, $CDCl_3$) of 5-AMF

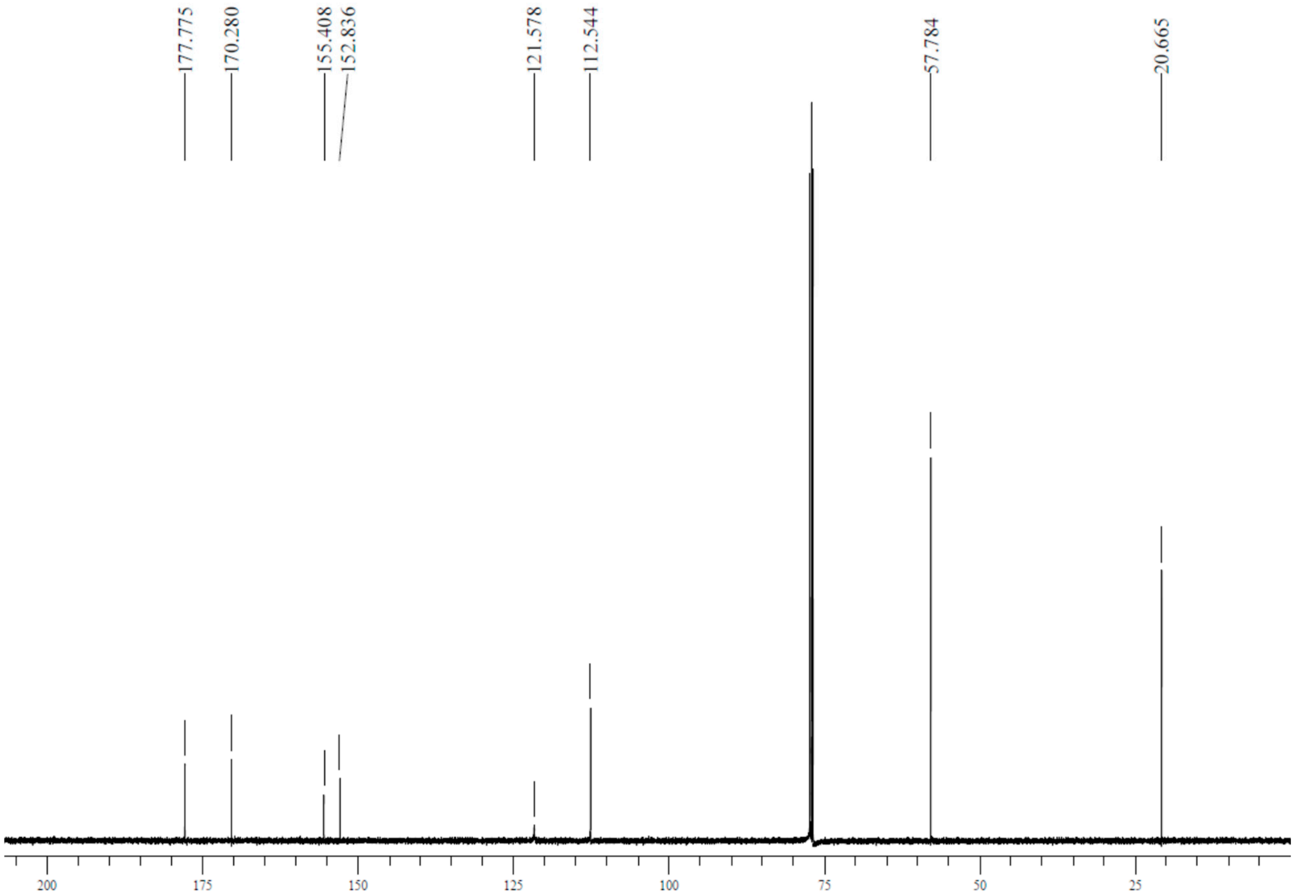

Figure S7. ^{13}C-NMR (125 MHz, $CDCl_3$) of 5-AMF

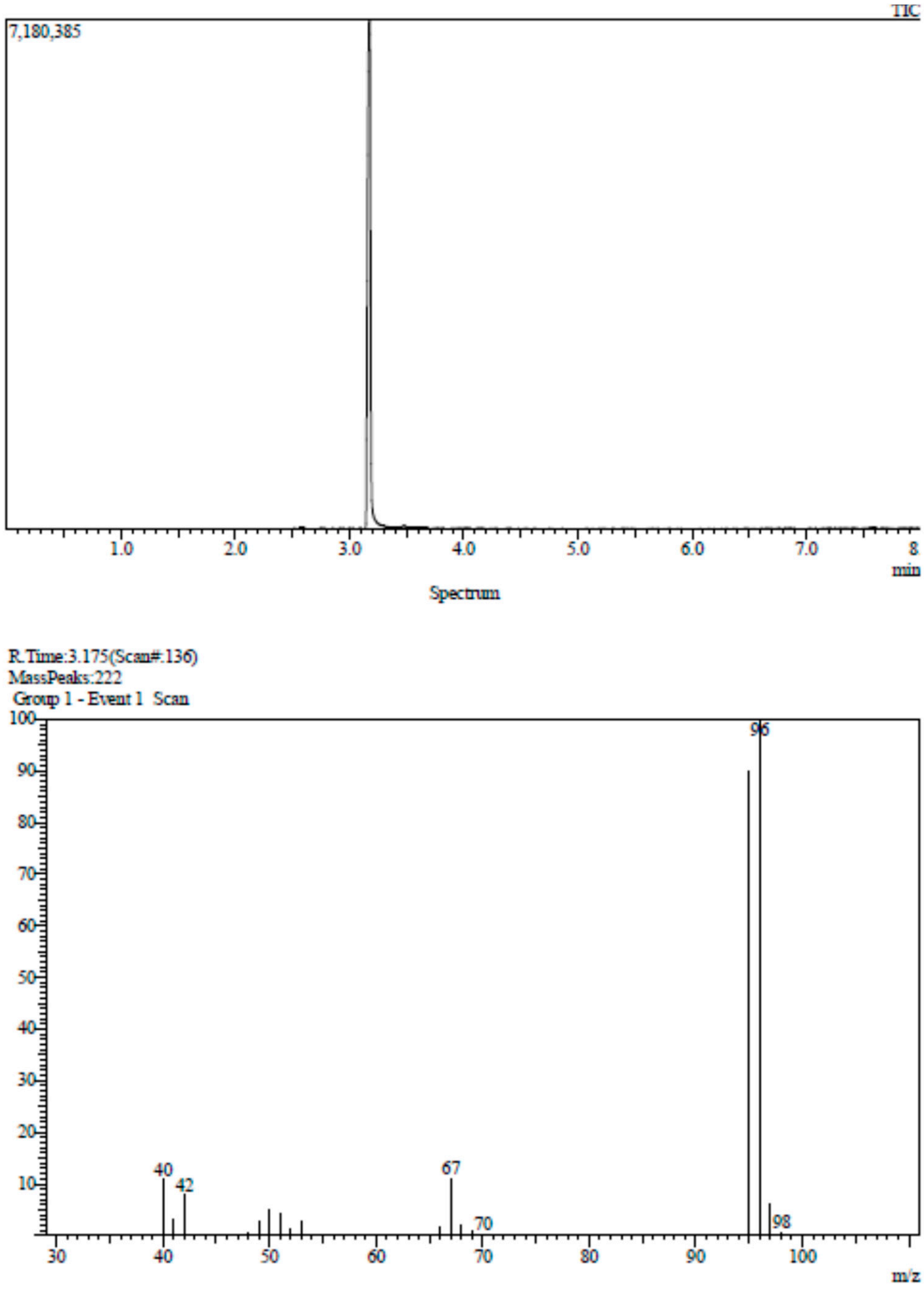

*Figure S8***.** GC/MS of furfural

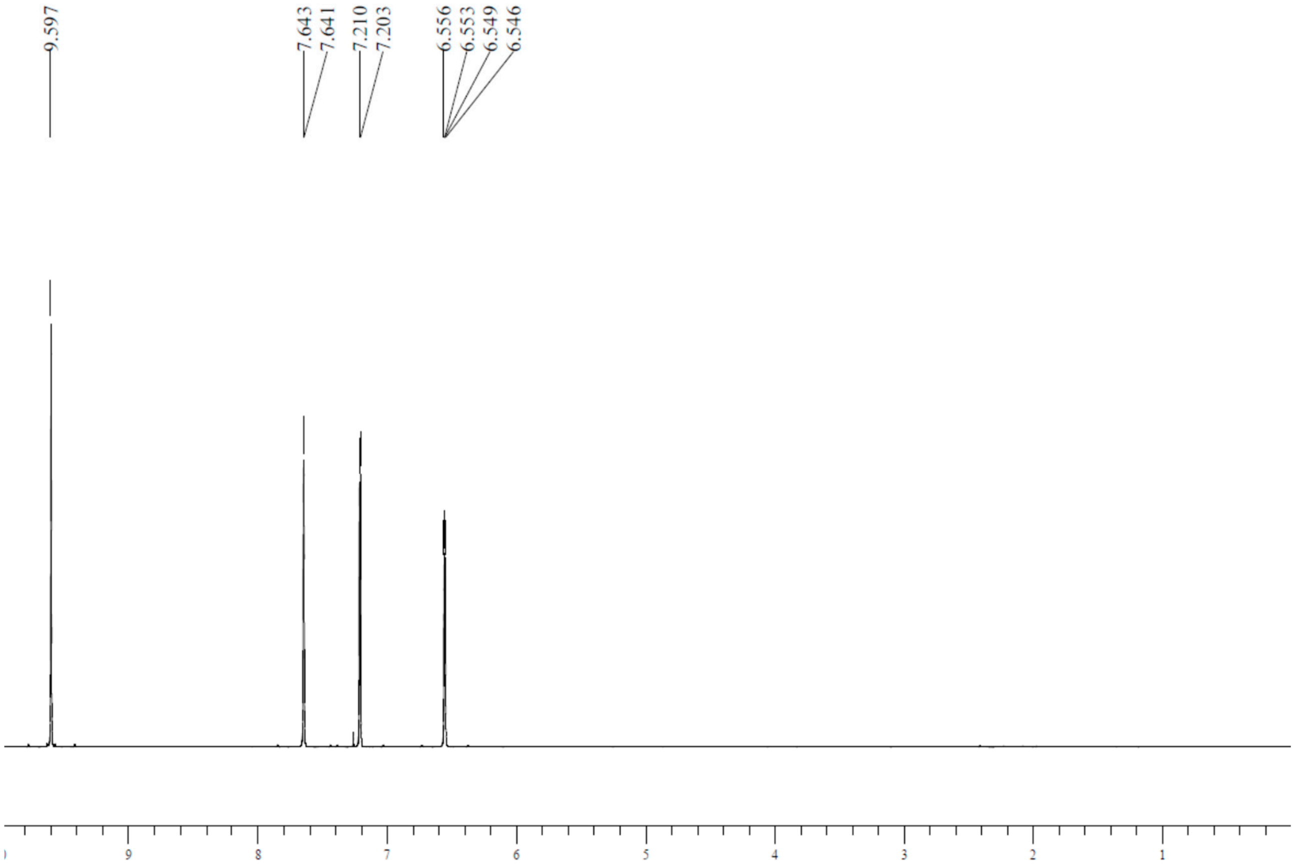

*Figure S9***.** ^{1}H-NMR (500 MHz, $CDCl_3$) of furfural

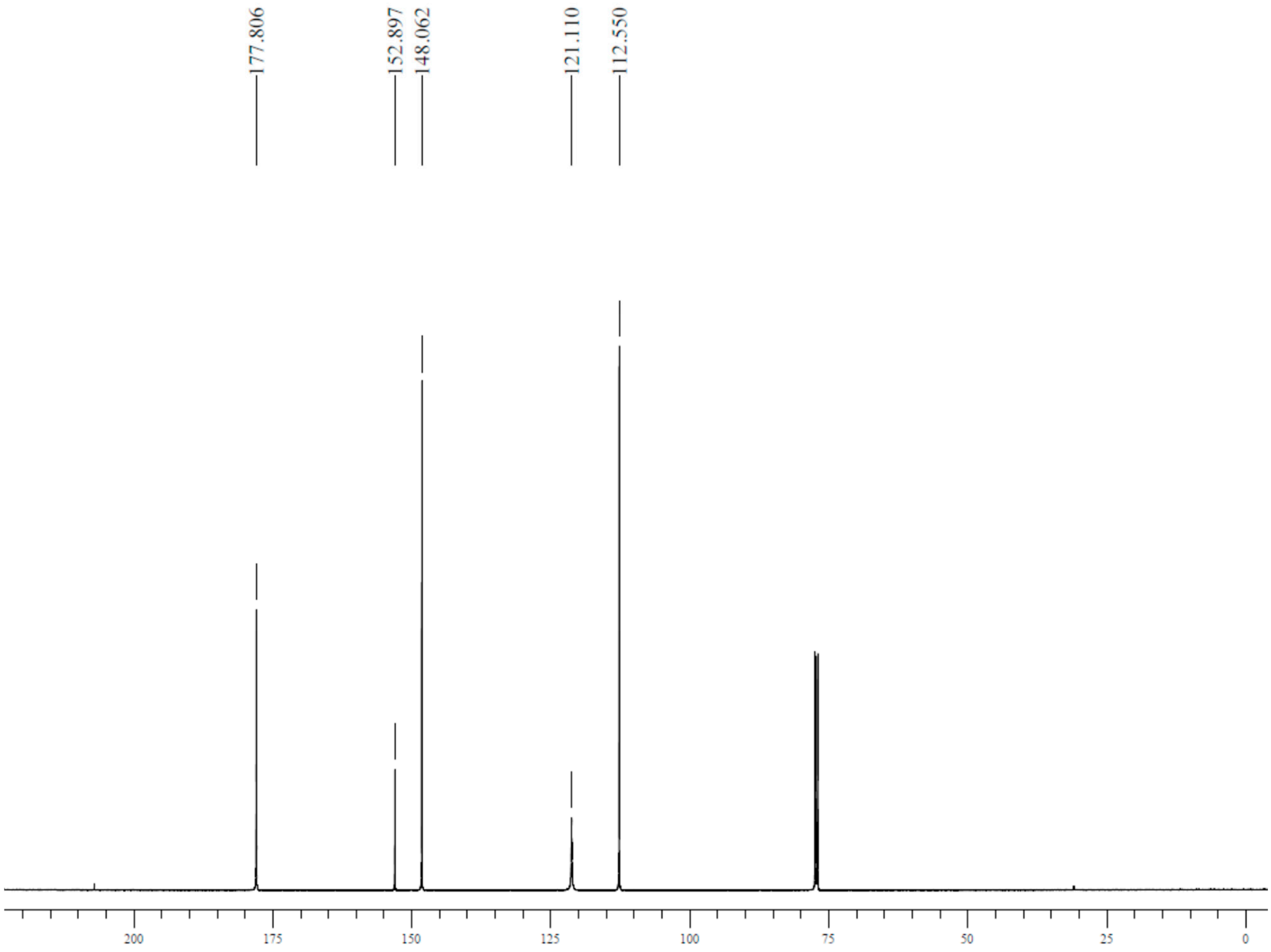

Figure S10. ^{13}C-NMR (125 MHz, $CDCl_3$) of furfural

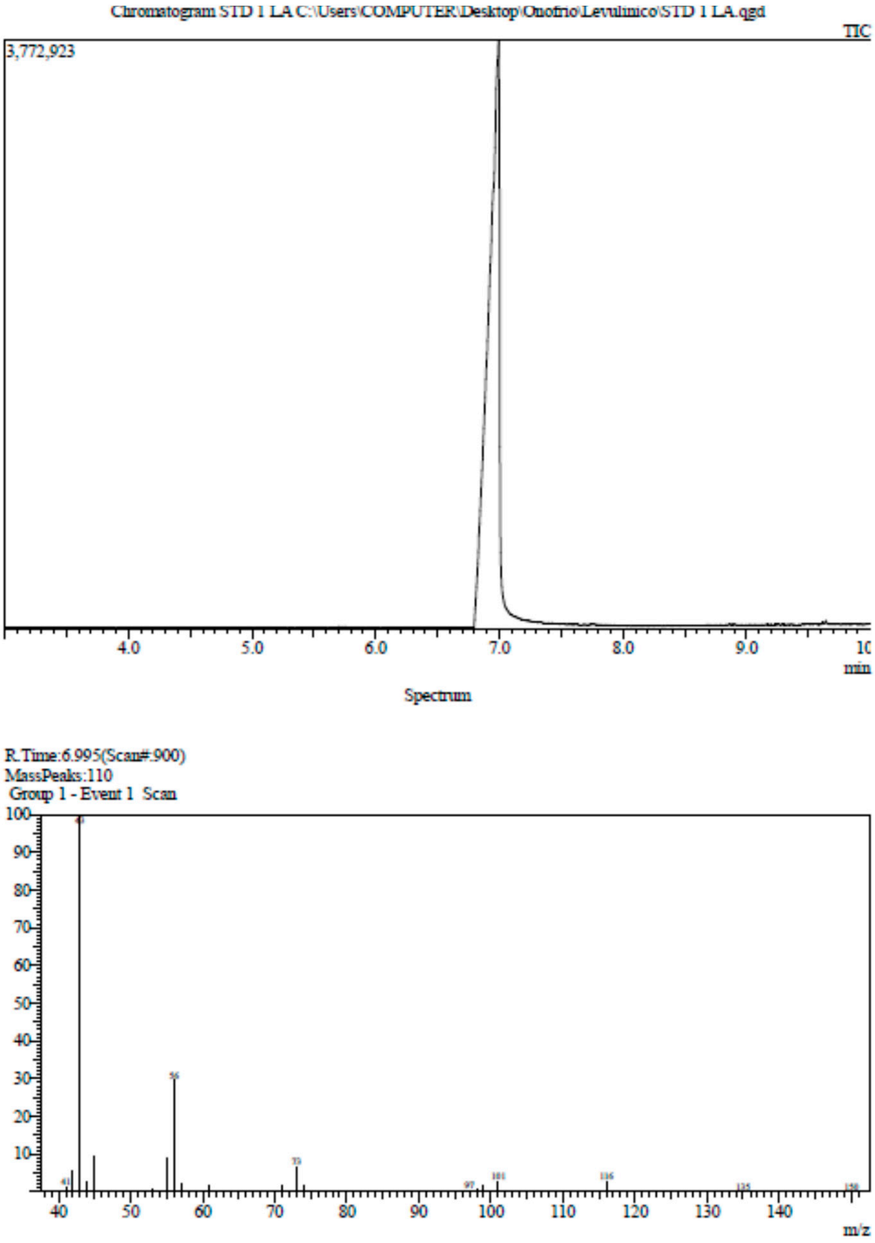

*Figure S11***.** GC/MS of Levulinic Acid

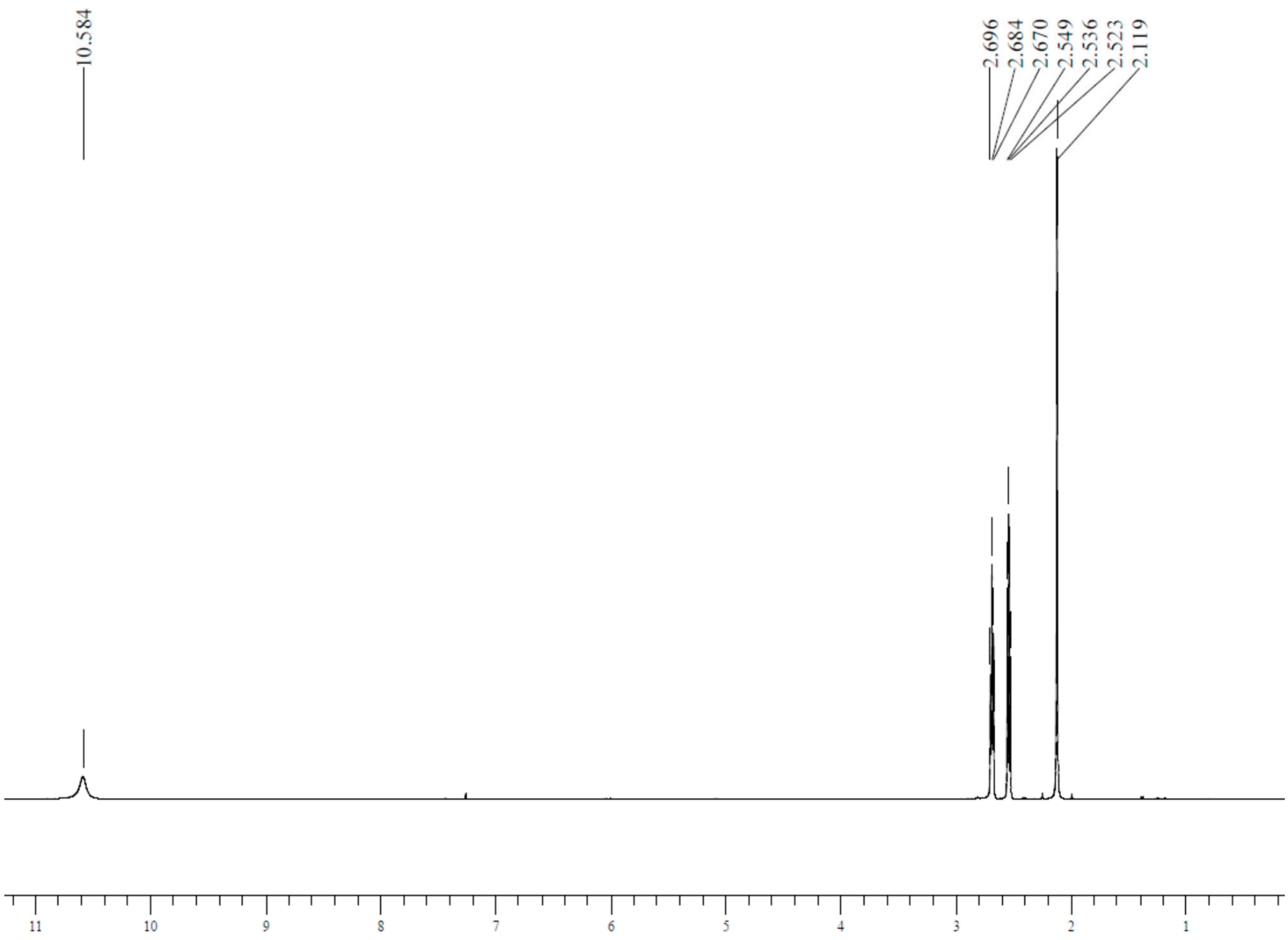

Figure S12. ^{1}H-NMR (500 MHz, $CDCl_3$) of Levulinic acid

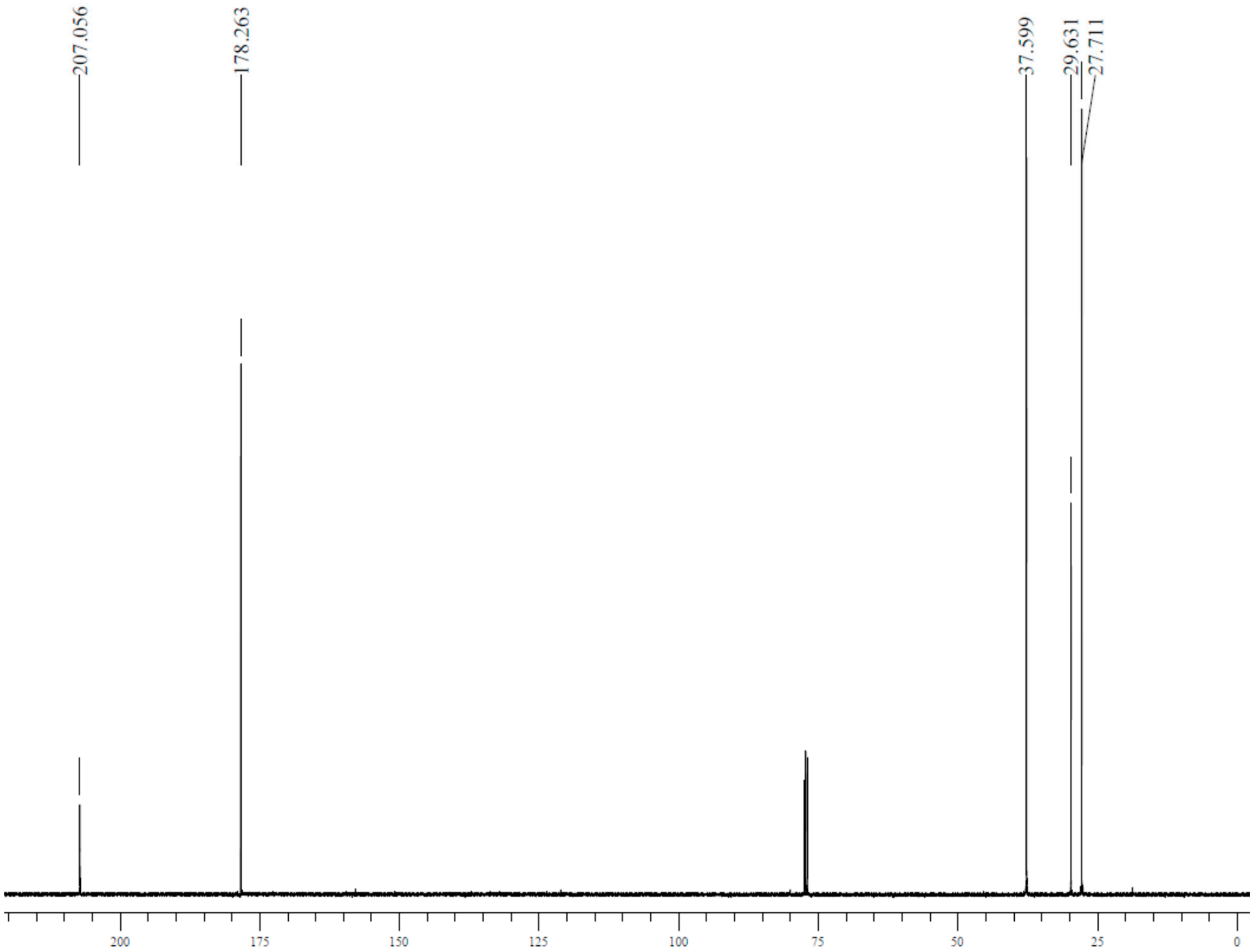

Figure S13. ^{13}C-NMR (125 MHz, $CDCl_3$) of Levulinic acid

Calibration curve for determination of LA

To evaluate yields of levulinic acid, a calibration curve was prepared by means of 4 standard solutions of pure LA into ethyl acetate at different concentration. GC-MS areas were collected (50.0 °C x 1 min to 150.0 rate 25 °C/min, to 170 °C rate 20 °C/min, to 250 rate 80 °C x 20 min) for each solution by means of three replicates as reported in Figure S14.

Standard soln.	C (% w/w)	Areas	SD
STD #1	0.2613	3,834,648	180,601
		3,583,892	
		3,934,418	
STD #2	0.5195	7,493,909	250,024
		7,027,383	
		7,104,758	
STD #3	0.7525	11,703,311	173,357
		11,589,283	
		11,362,738	
STD #4	0.9840	15,738,782	225,755
		15,552,728	
		15,289,478	

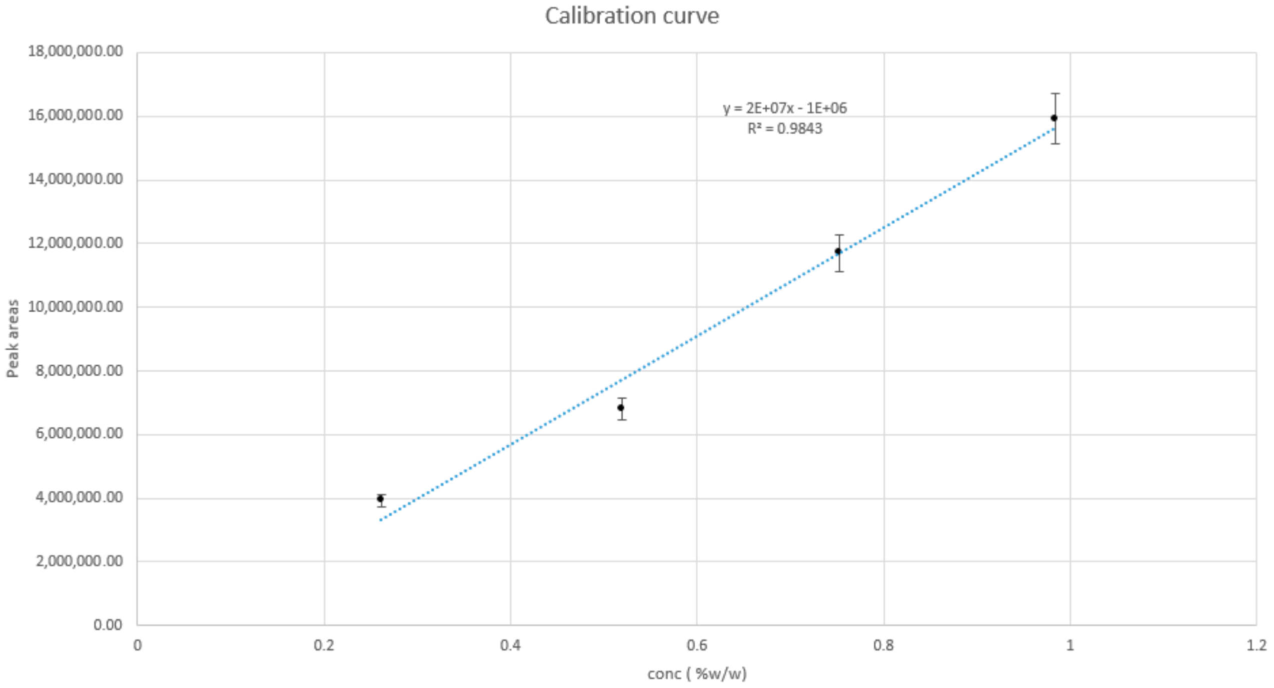

Figure S14. Calibration curve for evaluating yields of Levulinic Acid.

Calibration curve for determination of 5-AMF,5-HMF and furfural.

To evaluate yields of HMF,AMF and furfural, a calibration curve was prepared by means of 4 standard solutions of pure 5-HMF into ethyl acetate at different concentration. GC-MS areas were collected (50.0 °C x 1 min to 150.0 rate 25 °C/min, to 170 °C rate 20 °C/min, to 250 rate 80 °C x 20 min) for each solution by means of three replicates as reported in Figure S15

Standard soln.	C (% w/w)	Areas	SD
STD #1	0.18	5,107,856	274,720
		5,333,207	
		4,786,565	
STD #2	0.3737	10,753,171	926,449
		12,548,654	
		11,254,545	
STD #3	0.64	21,002,145	597,462
		21,652,123	
		20,458,784	
STD #4	0.8705	31,547,874	102,000,8.4
		32,555,875	
		33,587,844	

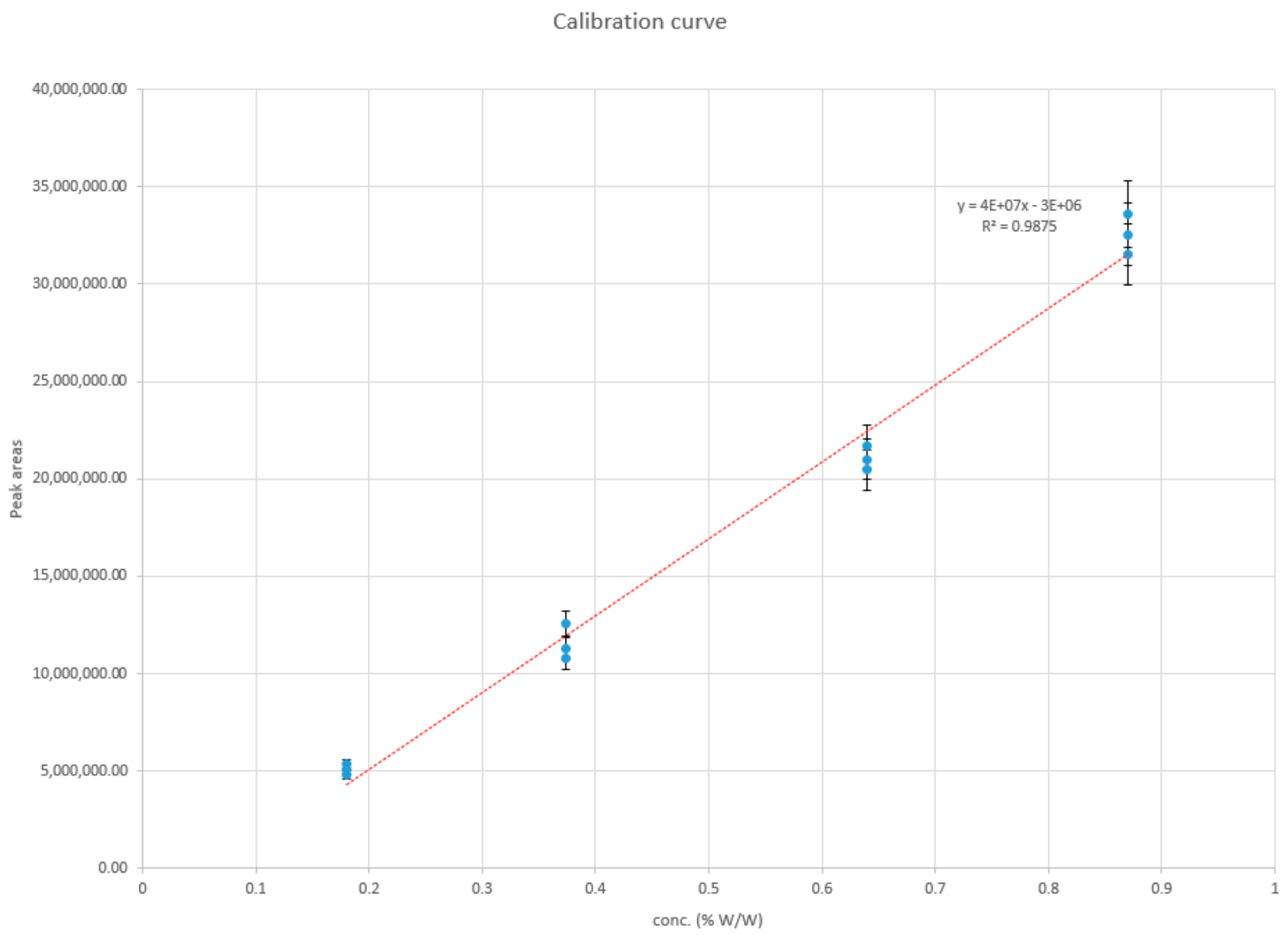

Figure S15. Calibration curve for evaluating yields of 5-HMF, furfural and 5-AMF.
